# A theory-informed, process-oriented Resident Scholarship Program

**DOI:** 10.3402/meo.v21.31021

**Published:** 2016-06-14

**Authors:** Satid Thammasitboon, John B. Darby, Amy B. Hair, Karen M. Rose, Mark A. Ward, Teri L. Turner, Dorene F. Balmer

**Affiliations:** 1 Department of Pediatrics, Center for Research, Innovation and Scholarship in Medical Education, Baylor College of Medicine, Houston, TX, USA; 2 Department of Pediatrics, Baylor College of Medicine, Houston, TX, USA; 3 Department of Pediatrics, Center for Clinical Effectiveness, Baylor College of Medicine, Houston, TX, USA; 4 Department of Pediatrics, Perelman School of Medicine, University of Pennsylvania, Philadelphia, PA, USA

**Keywords:** scholarly activity, residency training, research, self-directed learning, self-determination theory

## Abstract

**Background:**

The Accreditation Council for Graduate Medical Education requires residency programs to provide curricula for residents to engage in scholarly activities but does not specify particular guidelines for instruction. We propose a Resident Scholarship Program that is framed by the self-determination theory (SDT) and emphasize the process of scholarly activity versus a scholarly product.

**Methods:**

The authors report on their longitudinal Resident Scholarship Program, which aimed to support psychological needs central to SDT: autonomy, competence, and relatedness. By addressing those needs in program aims and program components, the program may foster residents’ intrinsic motivation to learn and to engage in scholarly activity. To this end, residents’ engagement in scholarly processes, and changes in perceived autonomy, competence, and relatedness were assessed.

**Results:**

Residents engaged in a range of scholarly projects and expressed positive regard for the program. Compared to before residency, residents felt more confident in the process of scholarly activity, as determined by changes in increased perceived autonomy, competence, and relatedness. Scholarly products were accomplished in return for a focus on scholarly process.

**Conclusions:**

Based on our experience, and in line with the SDT, supporting residents’ autonomy, competence, and relatedness through a process-oriented scholarship program may foster the curiosity, inquisitiveness, and internal motivation to learn that drives scholarly activity and ultimately the production of scholarly products.

Residency programs accredited by the Accreditation Council for Graduate Medical Education (ACGME) must provide curricula designed to advance residents’ knowledge of the basic principles of research and opportunities for residents to participate in scholarly activity (defined as taking a systematic approach to a question or to a project) (1, 2). To this end, residency programs have created experiential learning opportunities with the expectation that residents produce a scholarly product, typically a peer-reviewed abstract, manuscript, or presentation (3–10). Learning opportunities are often situated in scholarship programs that enforce timelines, provide oversight, and offer competitive awards, all for the purpose of motivating residents to ‘produce’ a scholarly product. Surely, a scholarly product can serve as evidence of participating in scholarly activity. However, we propose that residents are well served by scholarship programs that focus on the scholarly process and not on the scholarly product. First, a process-oriented scholarship program may provide a foundation upon which residents can produce scholarly products. Second, a process-oriented program is widely applicable across different content domains. Third, a process-oriented program may foster curiosity, inquiry, and internal motivation to learn that drives engagement in scholarly activity and, ultimately, the production of scholarly products (11). This production of scholarly products, when made available to others in the field and judged by peers to be a valuable contribution, is scholarship (2).

This trend article describes the process-oriented Resident Scholarship Program, developed in the Department of Pediatrics at Baylor College of Medicine in Houston, Texas, USA. It begins with a review of the theoretical framework that guides the Resident Scholarship Program, chronicles program development, and recounts preliminary program evaluation findings. It ends with a brief discussion of how, based on its theoretical framework, the program has had positive effects on residents.

## Background

In 2012, residency program leadership conducted a needs assessment to ascertain residents’ understanding of, and interest in, scholarly activity and scholarship. The needs assessment revealed that 1) residents valued pursuing scholarly activities that aligned with their personal interests, 2) many residents had participated in research before entering residency but were not currently engaged in any scholarly activities, and 3) residents tended to perceive scholarship as research and discount other types of scholarship (e.g., innovations in teaching, advocacy projects).

The following year, the program directors collaborated with the principal investigator to create a scholarship program that would address issues identified by the needs assessment and fulfill the ACGME mandate. The principal investigator formed an oversight committee consisting of an education researcher, a quality improvement specialist, and a faculty member interested in education. The oversight committee members acted under the auspices of the residency program to develop, implement, and evaluate a scholarship program for pediatric residents that spanned their three years of training.

The oversight committee members (hereafter referred to as we) drafted initial program aims after reviewing the literature and speaking to colleagues who administered scholarship programs in other residencies. As much as possible, we capitalized on existing educational activities; for example, we included lectures on research design and scientific writing in the noon conference curriculum. With the support of residency program directors, the Vice-Chair for Education, and the Chair of Pediatrics, we secured a 4-week required rotation for residents’ scholarly activity in the second year.

In the process of developing the Resident Scholarship Program, we familiarized ourselves with the self-determination theory (SDT). We embraced SDT as a theoretical framework, given its alignment with the aims and process-orientation of our program. Briefly, SDT purports that supporting three basic psychological needs – autonomy, competence, and relatedness – fosters intrinsic motivation to learn, that is, actions or behaviors driven by interest or enjoyment, not external pressures or rewards (12–14). Consistent with the SDT, *autonomy* entails choices and respecting alternative perspectives. *Competence* entails a reasonable level of challenge, and assistance in developing knowledge and skills to meet that challenge. *Relatedness* entails acknowledging human emotions and creating human connections.

With these psychological needs in mind, we revised our program aims to better align with the SDT framework. For example, and as displayed in Table 1, autonomy fit well with our aim to appeal to residents own career plans, but *relatedness* led us to think about ways to engage the larger pediatric faculty in residents scholarship. As also displayed in Table 1, we used the SDT to guide program evaluation, recognizing that theory-informed evaluation could go beyond measuring the achievement of program aims and shed light on how aims were achieved (15).

**Table 1:** Resident Scholarship Program aims and program evaluation

	Program aim	Program evaluation
Autonomy	Develop an experiential scholarship curriculum that fosters self-directed learning and residents’ pursuit of 1) the careers they choose and 2) the scholarly activities that best fit those careers	Assess perceptions of autonomy via program evaluation survey Determine the range of scholarship pursued by residents
Competence	Build a cohort of residents with knowledge, skills, and attitudes necessary to effectively engage in scholarship throughout their careers	Assess perceptions of competence via program evaluation survey Review reflective summaries Track completion of scholarly projects via follow-up query
Relatedness	Create a community of scholars—locally and beyond—in which collaborative scholarly activity and mentorship can thrive	Assess perceptions of relatedness via program evaluation survey

## Program description

We set forth intended learning outcomes for program components. As displayed in Table 2, learning outcomes align with at least one of the STD's basic psychological needs. In the following section, we describe program components; one component, participation in the Resident Scholarship Program core lecture series, extends across all 3 years.

**Table 2:** Intended learning outcomes for the Resident Scholarship Program, as they align with the self-determination theory

		Self-determination theory		
	
Intended learning outcomes		Autonomy	Competence	Relatedness
First year	Grasp a broad definition of scholarship fostering the choice of a scholarly project that aligns with resident's career interest	x		
	Select a project and a project mentor	x		x
	Develop a scholarly project, with input from project mentor		x	x
	Participate in core lecture series		x	
Second year	Participate in the 4-week required scholarly activity rotation (call free, vacation free), supervised by project mentor	x	x	
	Seek other learning opportunities, related to scholarly project from project mentor	x	x	
	Summarize and discuss resident's process of scholarship in reflective session		x	x
	If desired, and as time permits, continue to work on scholarly project	x	x	
	Participate in core lecture series		x	
Third year	If desired, and as time permits, continue to work on scholarly project	x	x	
	If desired, complete a 4-week scholarship elective	x		
	If desired, present at the Resident Scholarship Day, or another local, regional, national or international venue	x		x
	Participate in core lecture series		x	

### Year one

We designed the first year of the program to be an introduction to scholarship. We secured time early in residents' first year of training to introduce them to Boyer's (16) broad definition of scholarship, that is, discovery, integration, teaching, and application. We encouraged first-year residents to explore potential projects that piqued their interests and/or aligned with their envisioned career paths. We asked them to identify a project mentor by the end of the first year; residents could consult a faculty member who serves as a scholarship champion to help find a project mentor and explore a potential project.

### Year two

We designed the Resident Scholarship Program to be largely experiential in the second year of residency. All second-year residents must complete a 4-week, scholarly activity rotation. In this rotation, residents have protected time to work on their scholarly project under the supervision of their project mentor. Residents also have opportunity to explore project-related learning opportunities (e.g., attend committee meetings, complete online modules).

We encourage residents to use their scholarly activity rotation to delve into the scholarly process rather than concentrate on having a product by the end of the rotation. We use Glassick's criteria (17), outlined in a reflective summary, to guide residents in the scholarly process (Table 3). We review residents’ reflective summaries at the end of the rotation and probe for more information during an informal, end-of-rotation, reflective session. We invite project mentors, scholarship champions, residency program directors, and other residents to attend these sessions.

**Table 3:** Reflective summary for the Resident Scholarship Program

Glassick's criteria	Explanation	Response
Clear goals	What was your purpose/goal? Was it clear? Was it realistic?	
Adequate preparation	How did you gain an understanding of the literature? How did you gain skills and access resources you needed?	
Appropriate methods	How did you determine which methods to use to meet your purpose/goal? Did you find your methods effective?	
Significant results	Did you achieve your purpose? If not, where you in the process? If so, how do you suppose your results will inform the topic/area you studied?	
Effective communication	How do you plan to present your work? How will you ensure your message is clear, organized, and sound?	
Reflective critique	What feedback have you gotten about your project? How have you used feedback to improve?	

### Year three

We designed the third year of the program to be driven by third-year residents’ desire to bring their scholarly project to completion, or to engage in another scholarly project. We allow third-year residents to devote an elective rotation to their scholarly project and/or participate in the annual, department-wide Resident Scholarship Day.

## Program evaluation

As stated previously, we used the STD to guide program evaluation (Table 2). In this section, we share how we collected formative and summative evaluation data.

### Formative

We conducted formative evaluation in the spring of 2014. We interviewed four project mentors and asked residents who completed the scholarly activity rotation for feedback about the scholarship program. Based on that feedback, we added the end-of-rotation reflective sessions described above. The reflective sessions continue to be a means of collecting ongoing formative feedback about the program from residents, project mentors, and residency program directors. To better appreciate the perspective of project mentors, we asked one of them (AH) to join our oversight committee in 2014.

### Summative

We catalog and categorize residents’ scholarly projects by Boyer's types of scholarship. We get a general sense of residents’ engagement by reviewing their reflective summaries. We administer an online program evaluation survey every 6 months; retrospective pre-post items ask residents directly about gains in confidence in the processes of scholarly activity and indirectly about their sense of autonomy, competence, and relatedness. We also include several items asking specifically about how residents forged relationships with their project mentor.

For this report, we applied descriptive statistics to evaluation data. We used McNemar's test to compare (pre- vs. post-rotation) proportions of residents responding to survey items related to autonomy, competence, and relatedness. Our program evaluation has been approved by the IRB at Baylor College of Medicine.

## Preliminary evaluation findings

In the following section, we share evaluation data that we planned to collect as well as evaluation data that we did not plan to collect but view as evidence of the Resident Scholarship Program achieving its aims.

### Planned

Based on our catalog of scholarly projects for 2013–2014 and 2104–2015 academic years, and our review of reflective summaries, residents engaged in a variety of scholarship: discovery (52%, e.g., conducting hypothesis-driven research on antibiotic usage); application (30%, e.g., leading advocacy committee for anti-human trafficking); teaching (30%, e.g., preparing a curriculum for the underserved rotation); and integration (11%, e.g., presenting the results of a clinical quality improvement project designed to reduce medication errors).

Data from our program evaluation survey for 2013–2014 and 2014–2015 indicated that residents gained a broader understanding of scholarship as a result of the Resident Scholarship Program, with 96% (48/50), 98% (49/50), and 93% (46/50) seeing application, teaching, and integration, respectively, as scholarship. Nearly all residents reported positive regard for the program, with 68% rating the overall quality as ‘excellent’ or ‘outstanding’ and 30% as ‘good’. Open-ended feedback supported numeric ratings. For example, one resident commented, ‘The scholarship program is a phenomenal opportunity during residency. Please continue with this program as it is a unique opportunity for us work in an area of interest’. Another remarked,

“It was a very valuable learning experience to pursue a scholarly activity without competing clinical duties for a full block. And being able to complete a (scholarly) project during residency is intrinsically rewarding.”

Significantly more residents felt confident (pre- vs. post-rotation, *p*<0.05, McNemar's test) in selecting a project that aligns with their career interests (proxy for autonomy; 18% vs. 67%), directing their own learning as it related to their scholarly project (proxy for competence; 44% vs. 89%), disseminating scholarship (proxy for competence; 13% vs. 44%), collaborating around scholarly pursuits (proxy for relatedness; 31% vs. 73%), and establishing a mentoring relationship (proxy for relatedness; 3% vs. 91%). Open-ended feedback also supported the findings. For example, one resident commented, ‘I liked the independence this rotation gives and the protected time to work on a project of our choosing [autonomy]’. Another reported,

“I never participated in these things (scholarship) during college or medical school because I thought they were boring. But this month really gave me the confidence that I can write a paper that other people might care about [relatedness]. At this point I would be far more likely to write up something in the future than I have ever been in the past [competence].”

### Unplanned

Despite our process-orientation, we are encouraged that many residents continue to engage in scholarly activity in their final year of training. Of the 72 third-year residents who have responded to our follow-up queries in 2014 and 2015 (representing 92% of program participants), 58 (80%) reported either completing their project with recent dissemination (publication, presentation, or implementation) or planning dissemination during the coming year.

Residents’ enthusiastic regard for the Resident Scholarship Program caught the attention of the Vice-Chair for Education and the Chair of Pediatrics, resulting in funding for resident travel to present their scholarly projects, for Resident Scholarship Day, and for part-time administrative assistance. In addition, we have disseminated work about the Resident Scholarship Program at several national venues. Locally, our program received an Educational Scholarship Award, funded by Texas Children's Hospital, and was recognized with the Educational Innovation Award in the Department of Pediatrics at Baylor College of Medicine.

## Discussion

Based on our experience, and in line with the SDT, we believe that supporting residents’ autonomy, competence, and relatedness through a process-oriented scholarship program may foster the curiosity, inquisitiveness, and internal motivation to learn that drives scholarly activity and ultimately the production of scholarly products. Our use of the SDT as a program theory enables us to go beyond claims that our program worked and speculate about how it worked (15). First, residents choose their scholarly project and their project mentor, determine their own timeline for project completion, and decide if they want to participate in Resident Scholarship Day. Consistent with the SDT construct of autonomy, residents are free to do what piques their curiosity and what they consider relevant for their careers. Second, residents have opportunities to acquire knowledge, skills, and attitudes of scholarship that align with their career interests. Third, residents connect with like-minded scholars and gain a sense of belonging to groups with similar interests. Consistent with the SDT construct of competence, residents tap into resources they need to move their projects forward, and their careers in a desired direction. Consistent with the SDT construct of relatedness, residents engage with faculty and other trainees during monthly reflective sessions and at the Resident Scholarship Day.

On a practical level, SDT was a useful theoretical framework and aid for decision making as the Resident Scholarship Program evolved. For example, when deliberating about whether residents should be required to participate in Resident Scholarship Day, we chose to make it optional, thus supporting residents’ autonomy. When deliberating about whether residents could partner on a scholarly project, we decided in favor of partnership, thus supporting relatedness.

We acknowledge the limitations of our work, notably the lack of a comparison group and self-reported measures of autonomy, competence, and relatedness. The speculations we offer about our program outcomes are preliminary; in the future, we hope to determine if our appeal to residents’ curiosity, inquiry, and motivation to learn actually results in publications, presentations, and enduring materials.

Although others have used SDT to promote autonomy among pediatric residents (18), ours is a novel application of SDT as a theoretical framework for a scholarship program. The structure of our program, with its protected time in the second year of training, may be dissimilar from other training programs; nonetheless, using SDT as a theoretical framework has wide applicability. We believe that our use of SDT to guide program development and evaluation capitalizes on residents’ curiosity, inquisitiveness, and internal motivation to learn; fosters their engagement in scholarly activities that are meaningful and useful to them; and positions them well for producing scholarly projects.
